# Magneto-electrochemical biosensing for pathogen detection using nuclease-responsive nanohybrids

**DOI:** 10.1007/s00604-026-08087-3

**Published:** 2026-05-02

**Authors:** Gunes Kibar, Murat Kavruk, Frank J. Hernandez, Baris A. Borsa, Ali Dogan Dursun, Veli Cengiz Ozalp

**Affiliations:** 1 Department of Materials Science and Engineering, Micro Nano Particles (MNP) Research Group, Adana Alparslan Turkes Science and Technology University, Adana, 01250 Türkiye; 2 Vocational School of Health Services, Atilim University, Ankara, 06830 Türkiye; 3 Institute of Nanotechnology and Biotechnology, Istanbul University - Cerrahpasa, Istanbul, 34500 Türkiye; 4 Health Biotechnology Joint Research and Applications Center of Excellence, Istanbul, 34220 Türkiye; 5 Nucleic Acids Technologies Laboratory (NAT-Lab), Linköping University, Linköping, Sweden; 6 Department of Biomedical Engineering, Atlas University, Istanbul, 34408 Türkiye; 7 TECNUN, University of Navarra, San Sebastian, 20009 Spain; 8 IKERBASQUE, Basque Foundation for Science, Bilbao, Spain; 9 Centre for Laboratory Animal Breeding and Experimental Research (ATÜDEM), Atilim University, Ankara, Türkiye; 10 Home Care Services, Medicana International Ankara Hospital, Ankara, Türkiye

**Keywords:** POSS, *Streptococcus pneumoniae*, Electrochemical biosensor, Nuclease-based detection, Green synthesis, Emulsion polymerization, UV-polymerization, Superparamagnetic iron oxide nanoparticles

## Abstract

The development of sustainable and highly sensitive diagnostic platforms is critical for rapid pathogen identification and effective disease management. Here, a green, magneto-electrochemical biosensing strategy is reported for the selective detection of *Streptococcus pneumoniae* based on pathogen-specific nuclease activity. Uniform organic–inorganic hybrid polyhedral oligomeric silsesquioxane (POSS) nanoparticles were synthesized via an ultrafast UV-initiated emulsion polymerization within 5 min using an eco-friendly approach. The nanoparticles were sequentially functionalized by in situ deposition of superparamagnetic iron oxide nanoparticles and biomimetic polydopamine coating, enabling robust and high-density immobilization of nuclease-responsive oligonucleotide probes. The resulting PDA@SPION/POSS nanohybrids exhibit controlled size, preserved structural integrity, and strong superparamagnetic behavior, allowing efficient magnetic manipulation and electrochemical signal transduction. Upon exposure to *S. pneumoniae*, nuclease-mediated probe cleavage produces a pronounced electrochemical response, enabling label-free detection over a wide dynamic range (10^2^–10⁸ CFU mL⁻¹) with a detection limit of 10^2^ CFU mL⁻¹. High selectivity against non-target bacteria highlights the specificity of the enzymatic recognition mechanism. This work establishes a sustainable and amplification-free biosensing platform with strong potential for rapid clinical diagnostics.

**Graphical abstract:**

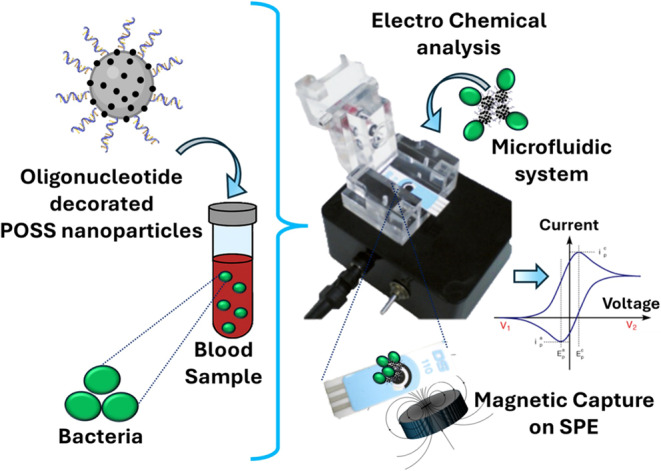

## Introduction

*Streptococcus pneumoniae* (*S. pneumoniae*) is a highly virulent bacterial pathogen and a leading cause of vaccine-preventable deaths worldwide. It is responsible for a wide range of invasive and non-invasive diseases, including pneumonia, meningitis, sepsis, otitis media, and sinusitis [1]. Among these, pneumonia remains the most prevalent and deadly, particularly in young children and older adults. Globally, the bacterium contributes to over 1.1 million deaths annually, with the highest burden observed in low- and middle-income countries [2]. In addition, the World Health Organization highlights *S. pneumoniae* as a priority pathogen due to its capacity for causing invasive diseases and its contribution to antimicrobial resistance [3]. *S. pneumoniae* is identified in various clinical samples; cerebrospinal fluid and blood are crucial for diagnosing invasive diseases like meningitis and sepsis, whereas sputum and nasopharyngeal swabs are used for pneumonia and epidemiological surveillance. Accurate identification is essential for guiding appropriate antibiotic therapy to ensure effective patient outcomes, which is critical in the face of rising antimicrobial resistance. Furthermore, detecting and serotyping the bacterium is vital for public health surveillance to monitor vaccine effectiveness and track the emergence of non-vaccine serotypes [4].

As the demand grows for faster and more precise ways to detect *S. pneumoniae*, researchers have turned to nanotechnology to enhance diagnostic tools [5]. Recently, biosensors using materials like gold nanoparticles and carbon-based nanocomposites have shown excellent sensitivity and selectivity. While some studies focused on detecting the key *S. pneumoniae* toxin, pneumolysin via lateral-flow sensor or an amperometric magnetoimmunosensor [5, 6], other studies utilized nanoparticles for detection of the bacteria itself [7]. In another design, glassy carbon electrode was utilized to decrease detection limits as low as 0.0022 ng/mL, roughly 600 bacterial cells, for enhancing the potential for real-world usage [8].

More complex systems are also emerging that can identify multiple pathogens at once. One recent study developed a magnetic biosensor capable of simultaneously detecting *S. pneumoniae* and *Klebsiella pneumoniae* using dye-labeled silica nanoparticles and a catalytic hairpin DNA reaction. This approach achieved over 96% accuracy with real patient samples, showing great promise for use in hospitals or field settings [9]. Building on this progress, our research group recently introduced a new biosensor platform that uses magnetic beads coated with DNA probes specifically designed to detect nuclease activity—a hallmark of pneumococcal infection. When these probes are cleaved by nucleases produced by the bacteria, the system provides a rapid and highly specific readout, offering a novel, label-free method for S. pneumoniae detection [10].

Magnetically assisted electrochemical biosensors (MAEB) have emerged as a promising solution for detecting pathogens quickly and accurately. These systems combine the selective capturing power of magnetic particles with the sensitivity of electrochemical measurements [11]. Typically, these platforms allow the specific capture of bacteria using bio-recognition elements, followed by redox signal generation via enzymatic or nanomaterial-based labels under the guidance of an external magnetic field. For example, Bu et al. (2020) successfully used ferrocene-loaded Cu₃(PO₄)₂ nanocomposites integrated with MBs to detect Salmonella typhimurium in milk, achieving excellent sensitivity [12]. Likewise, Zhu et al. (2022) reported the detection of Escherichia coli O157:H7 using HRP/Au@Pt-functionalized reduced graphene oxide on magnetic beads, with a detection limit of 91 CFU/mL [13]. These applications illustrate the powerful potential of MAEBs in pathogen diagnostics.

The strategy of utilizing bacterial nucleases via modified and optimized sequences for species-selective detection has been demonstrated previously [14–16]. In our previous study, the Fc-labelled P3 probe was validated on a planar gold-electrode format for electrochemical detection of *S. pneumoniae*-associated nuclease activity after standard bacterial culture preparation, and the electrochemical response was monitored over short probe–bacteria contact times (5–60 min) [10]. In the present work, we transfer the same nuclease-responsive probe chemistry to a magnetic nanoparticle-based MAEB configuration. Accordingly, this study evaluates the performance of the MAEB architecture for *S. pneumoniae* detection and the incubation-time requirements of the current platform.

## Materials and methods

### Materials

The hybrid monomer, methacryl-Polyhedral Oligomeric Silsesquioxane (M-POSS) cage mixture (MA-0735) was purchased from Hybrid Plastics Inc. An emulsifying agent sodium dodecyl sulphate (SDS), iron salts Iron(II) chloride tetrahydrate (FeCl2·4H2O), Iron(III) chloride hexahydrate (FeCl3·6H2O), the UV initiator 2-hydroxy-2-methylpropiophenone (Darocur 1173 or HMPP), ammonium hydroxide NH4OH (25% w/w) solution were obtained Sigma-Aldrich. Absolute ethanol was purchased from Merck A.G., Darmstadt, Germany. Distilled deionized (DDI) water used in all experiments was obtained by using Millipore/Direct Q-3UV. Dopamine hydrochloride (DOPA-HCl) was obtained from Sigma-Aldrich.

### POSS nanoparticle synthesis

**Fig. 1:**
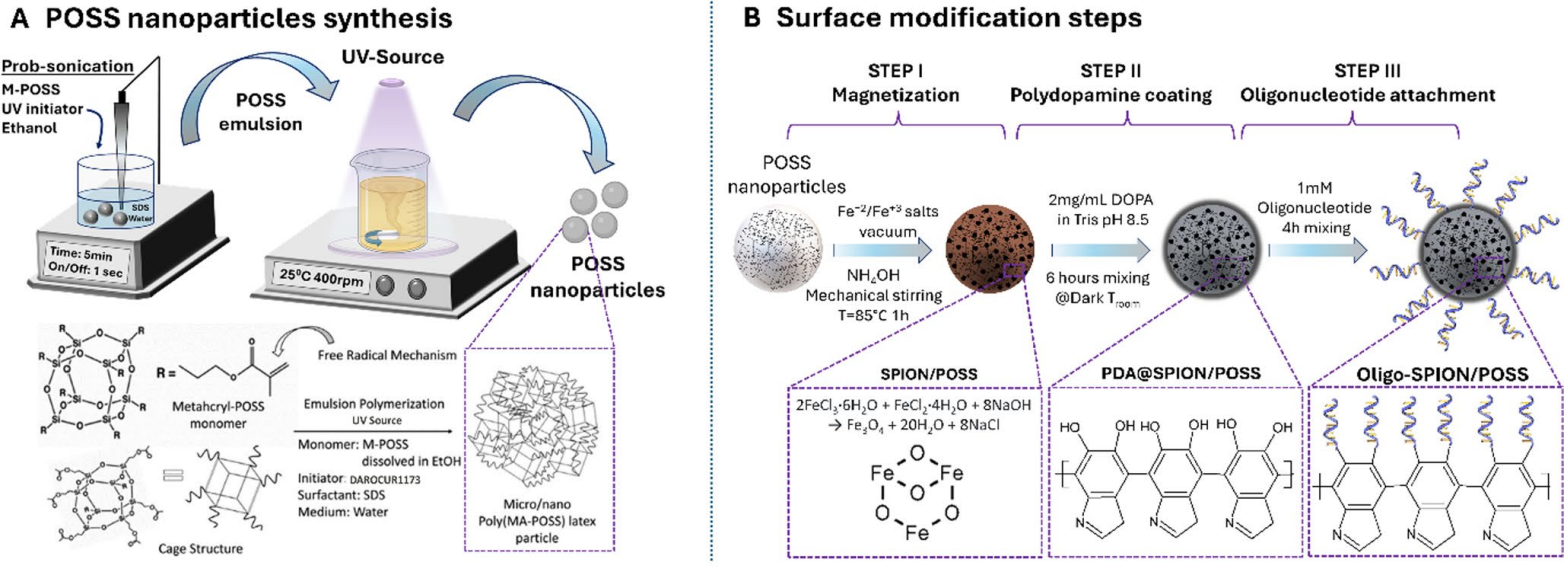
Schematic representation of (**A**) nanoparticle synthesis and (**B**) surface modification steps

The reaction medium was prepared via a two-phase system consisting of an aqueous and an organic phase [17]. The aqueous phase was composed of 0.25% (w/v) sodium dodecyl sulfate (SDS) dissolved in 10 mL of deionized water and was ultrasonicated in an ice bath for 5 min to ensure complete dissolution and dispersion. The organic phase was prepared by dissolving 50 mg of methacryl-functionalized polyhedral oligomeric silsesquioxane (M-POSS) and 0.02 mL of Darocur 1173 photo-initiator in 1 mL of absolute ethanol. For emulsion formation, the organic phase was slowly added to the aqueous phase under continuous ultrasonication for 5 min to generate a stable oil-in-water emulsion. Photopolymerization was carried out by exposing the emulsion to UV light (365 nm, 10 W LED) at a distance of 2 cm, under constant magnetic stirring for 5 min (Fig. 1a). Nanoparticle collection was achieved by centrifugation at 15,000 rpm for 2 min. The resulting white pellets were washed thoroughly with ethanol and deionized water several times to remove residual surfactant and unreacted components.

### In-situ SPION decoration on POSS nanoparticles

The surface decoration of poly(POSS) nanoparticles with superparamagnetic iron oxide nanoparticles (SPIONs) was carried out based on previously reported methods [18, 19], with slight modifications (Fig. 1b). To begin, 0.5 g of dried poly(POSS) nanoparticles were dispersed in 25 mL of deionized water under constant stirring to form a uniform suspension. In a separate step, iron precursors—0.14 g of FeCl₂·4 H₂O and 0.2 g of FeCl₃·6 H₂O—were dissolved in 5 mL of deionized water under a nitrogen atmosphere to minimize oxidation during the process [20].

The nanoparticle suspension was then heated to 85 °C using an oil bath while being mechanically stirred. To trigger the in-situ formation of Fe₃O₄ nanoparticles, 0.250 mL of ammonium hydroxide solution (25% w/w) was added to the mixture. A rapid color shift from brown to black was observed, indicating the successful formation of magnetite nanoparticles. The reaction was maintained at 85 °C for an additional 60 min to allow complete precipitation. Upon cooling to room temperature, the resulting magnetic poly(POSS) nanoparticles were isolated using a strong external magnet, washed several times with deionized water to remove residual reagents, and dried prior to further characterization.

### The biomimetic coating on SPION/POSS nanoparticles

POSS nanoparticles were coated with polydopamine following the method described by Kibar et al. [19–21]. Briefly, 0.25 mg of the synthesized POSS nanoparticles were dispersed in 10 mL of Tris buffer (pH 8.5) containing 2 mg/mL dopamine hydrochloride (DOPA-HCl, Sigma-Aldrich). The suspension was magnetically stirred for 6 h under dark conditions (Fig. 1b). During the reaction, the solution colour gradually changed from white to dark brown, indicating successful polymerization of dopamine. After coating, the PDA-modified POSS nanoparticles were collected by strong magnet and washed several times with deionized water. Finally, the particles were dried in a vacuum oven at 50 °C overnight.

### Probe attachment on PDA@SPION/POSS nanoparticles

The probe was the same as used in our previous study as “5’-Ferrocene- mUmUmUmU-fUfAfAfU-mUmUmUmU - Thiol − 3” (Fc-P3 probe) [10]. Thiol-groups can be conjugated spontaneously to the quinone groups on the PDA surface through Michael addition. The Fc-P3 probe (5 nmol) was first treated with 10 mM TCEP (Tris(2-carboxyethyl)phosphine) for 30 min and then the mixture was incubated with PDA@SPION/POSS nanoparticles (1 mg) 4 h in phosphate buffer (pH = 7.5) with 5 mM MgCl_2_ (Fig. 1b). Then the particles were collected via a strong magnet and washed with water several times. The oligomer concentration was analyzed via a Nanodrop spectroscopy (Thermo Fisher Scientific, USA) at 260 nm and the attached oligonucleotide probe amount was determined as follows

$$Probe\;attached=\;\frac{C{\left(probe\right)}_{initial}-C{\left(probe\right)}_{redidual}}{m\left(Nanoparticles\right)}\times Volume$$

where C(probe)initial and C(probe)residual denote the oligomer concentrations in the adsorption solution before and after nanoparticle incubation, respectively. m(nanoparticles) indicates the mass (mg) of the PDA@POSS nanoparticles used in adsorption and Volume (mL) is the total volume of the adsorption solution. The calculated value corresponds to the amount of oligomer bound per unit mass of nanoparticles.

### Characterization of nanoparticles

The morphological characteristics of the synthesized POSS nanoparticles were investigated using scanning electron microscopy (SEM) (Quanta 450, Akishima, Tokyo, Japan). For sample preparation, the nanoparticle suspension was homogenized by vortexing, and 2 µL of the dispersion was dropped onto double-sided conductive carbon tape mounted on a flat aluminum sample stub. The samples were air-dried for 5 min and subsequently sputter-coated with a 6 nm Au–Pt layer to enhance surface conductivity and prevent beam-induced thermal degradation during SEM analysis. Elemental composition and surface chemistry were further characterized by energy-dispersive X-ray spectroscopy (EDX) integrated within the same SEM system. The size distribution was analyzed in water via dynamic light scattering using Zetasizer Nano ZS (Malvern Instruments with SOP values of refractive index 1.46 and adsorption parameter 0.5).

Fourier-transform infrared spectroscopy (FTIR) (Thermo Scientific Nicolet™, USA) was employed to analyze the chemical bonding structures of the monomer and the resulting POSS nanoparticles. Spectra were acquired in the range of 4000–500 cm⁻¹ from samples prepared in powder form.

The crystalline structure of the POSS nanoparticles was examined using X-ray diffraction (XRD) analysis performed on a Rigaku Miniflex 600 diffractometer (Japan). Powdered samples were scanned under an accelerating voltage of 40 kV and a current of 15 mA, with a scanning rate of 2.000°/min and a step size of 0.020° over a 2θ range of 5° to 90°.

Magnetic properties of surface-functionalized POSS nanoparticles were evaluated using a vibrating sample magnetometer (VSM) (Cryogenic Limited, Model: PPM System, UK), providing insights into their superparamagnetic behavior post-modification.

### Sample preparation

**Fig. 2:**
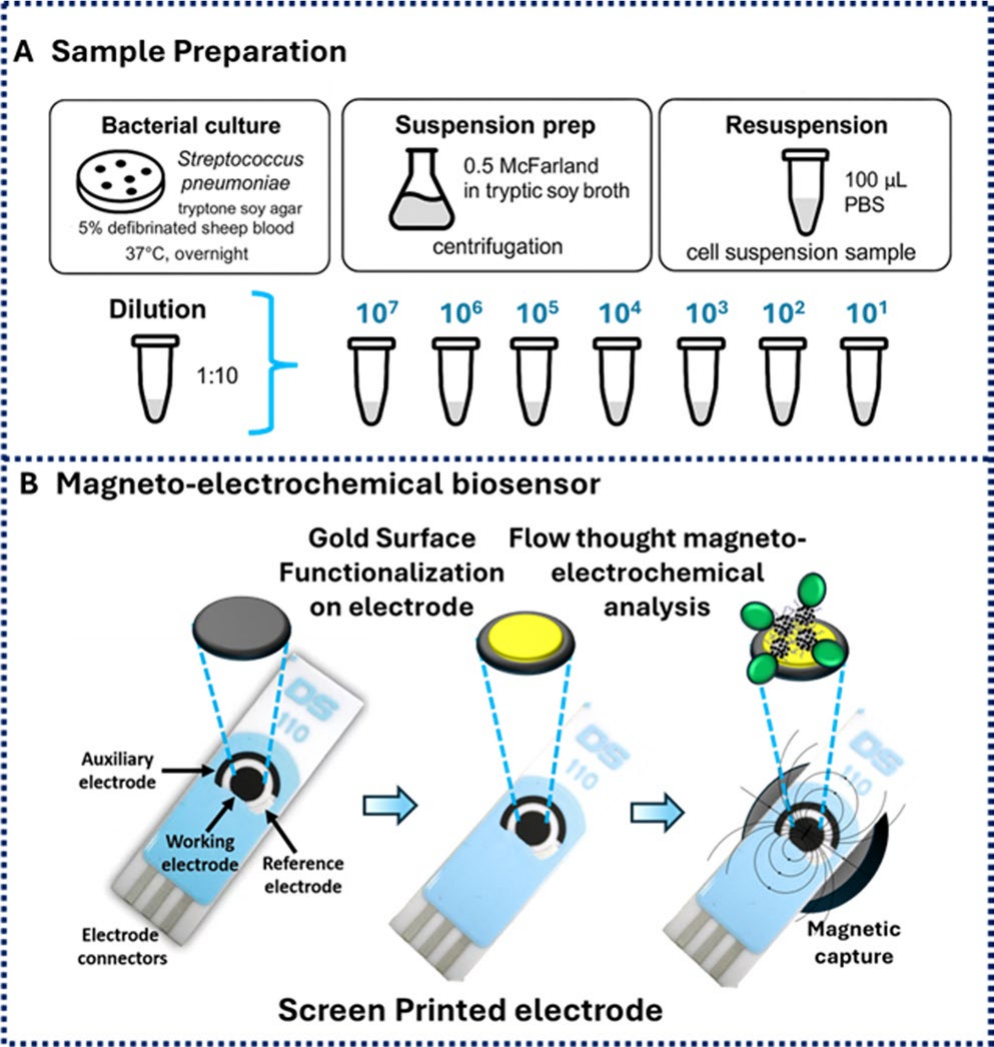
Schematic representation of (**A**) sample preparation and (**B**) magneto-electrochemical biosensor

**Fig. 3:**
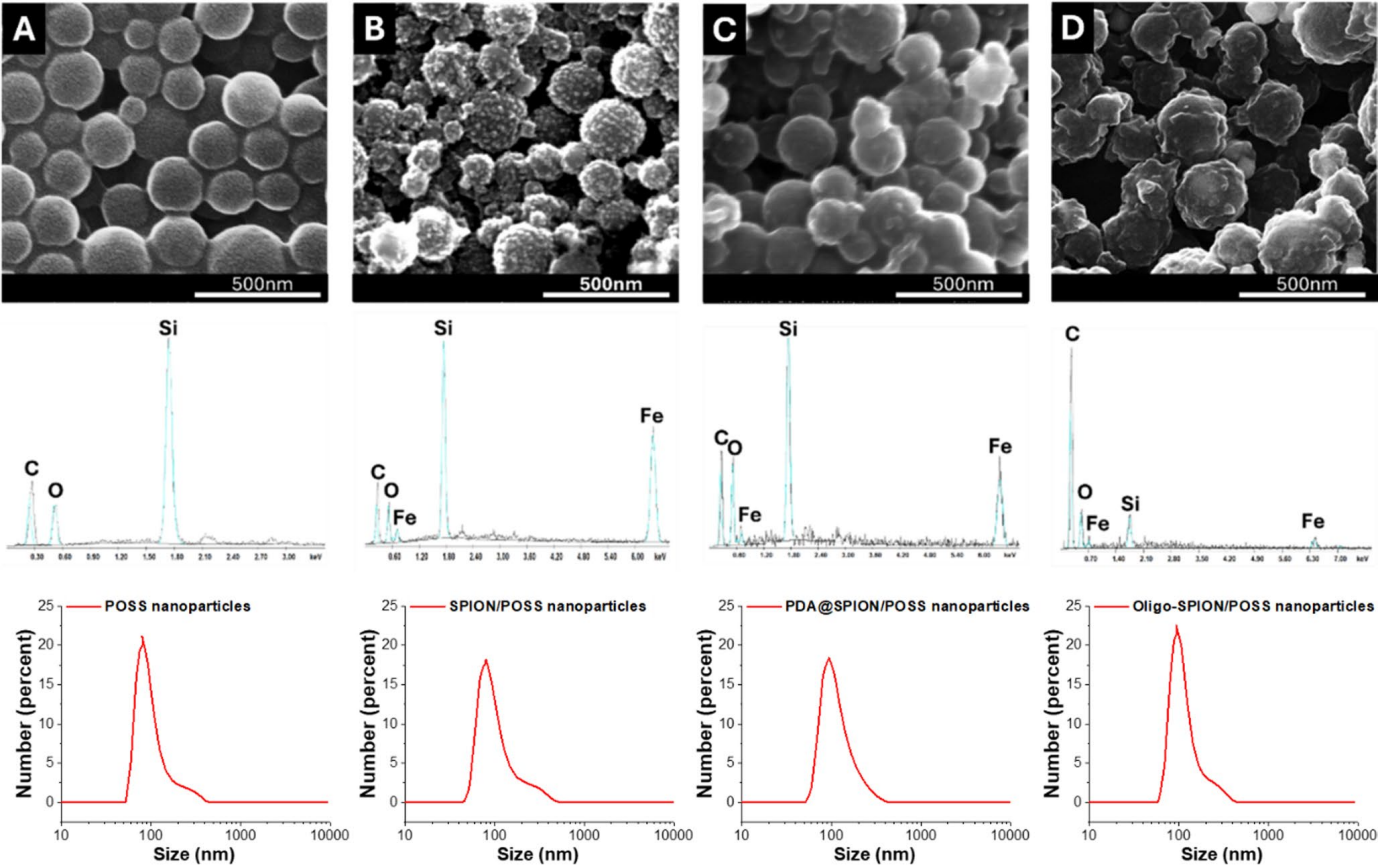
SEM images, energy-dispersive X-ray spectroscopy (EDX) spectra and DLS measuments of (**A**) POSS nanoparticles, (**B**) SPION/POSS Nanoparticles, (**C**) PDA@SPION/POSS nanoparticles, (**D**) Oligo-SPION/POSS nanoparticles

**Fig. 4:**
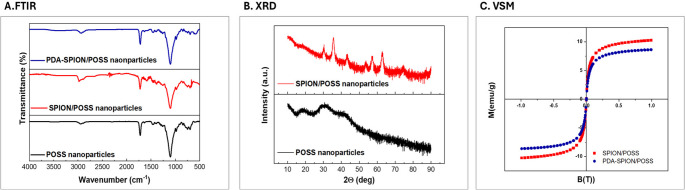
Characterization panel: (**A**) chemical structure: FTIR spectrum of POSS, SPION/POSS and PDA-SPION/POSS nanoparticles, (**B**) crystalline structure XRD analysis of POSS and SPION/POSS nanoparticles, (**C**) magnetic behaviour analysis VSM spectrum of SPION/POSS and PDA-SPION/POSS nanoparticles

**Fig. 5:**
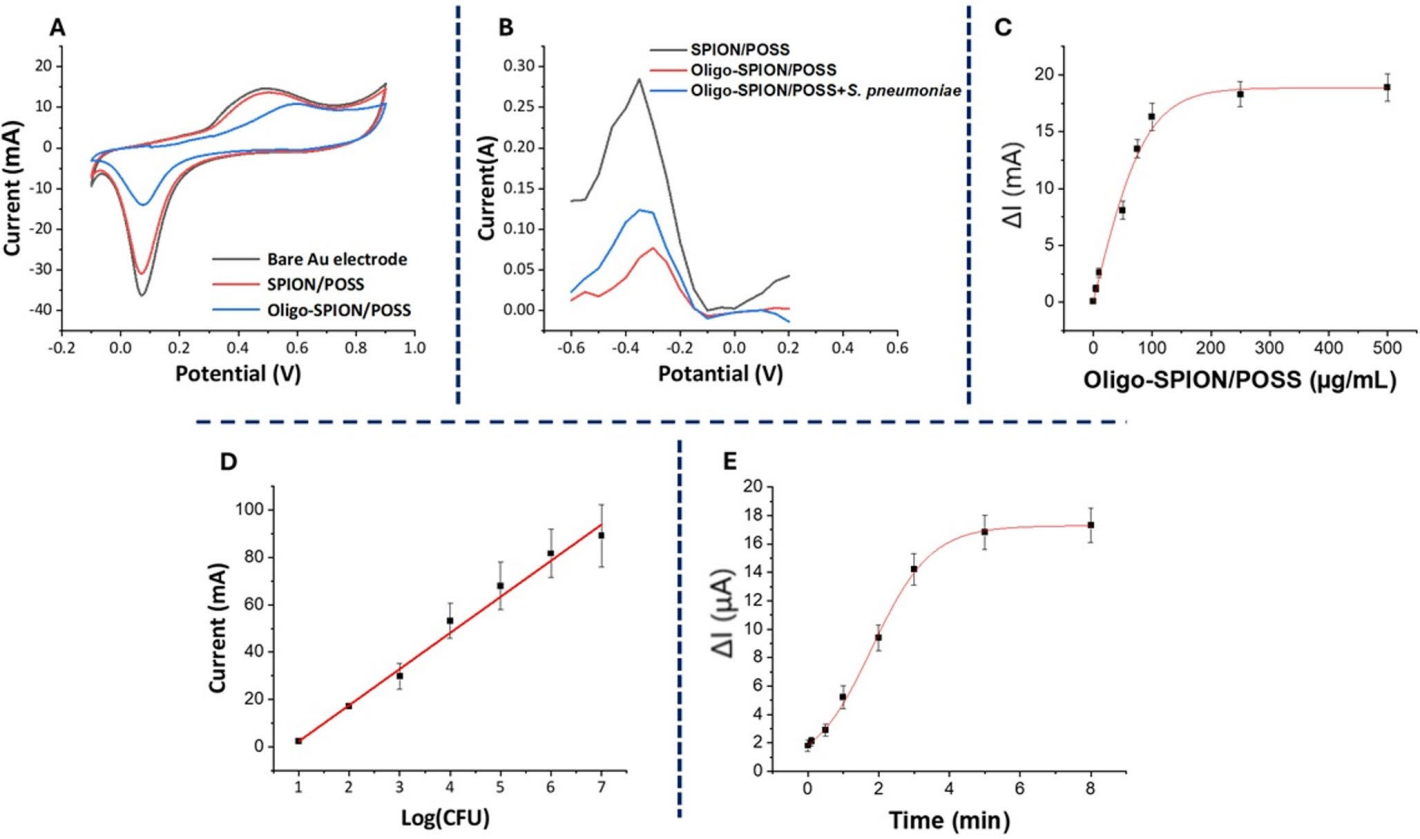
Electrochemical detection: (**A**) cyclic voltammograms; (**B**) SWV response of SPION/POSS (probe-free nanoparticle control), oligo-SPION/POSS (bacteria-free probe-coated control), and oligo-SPION/POSS after exposure to *S. pneumoniae*; (**C**) the effect of hybrid nanoparticle amount on the signal for 10⁵ CFU mL⁻¹ of *S. pneumoniae*; (**D**) sensitivity of electrochemical detection from 10¹ to 10⁸ CFU mL⁻¹ of *S. pneumoniae* cultures; and (**E**) incubation time during sample preparation reported as signal change, ΔI (µA), for 10² CFU mL⁻¹ bacteria and 100 µg hybrid nanoparticles. Data are presented as mean ± standard deviation from three independent experiments (*n* = 3)

**Fig. 6:**
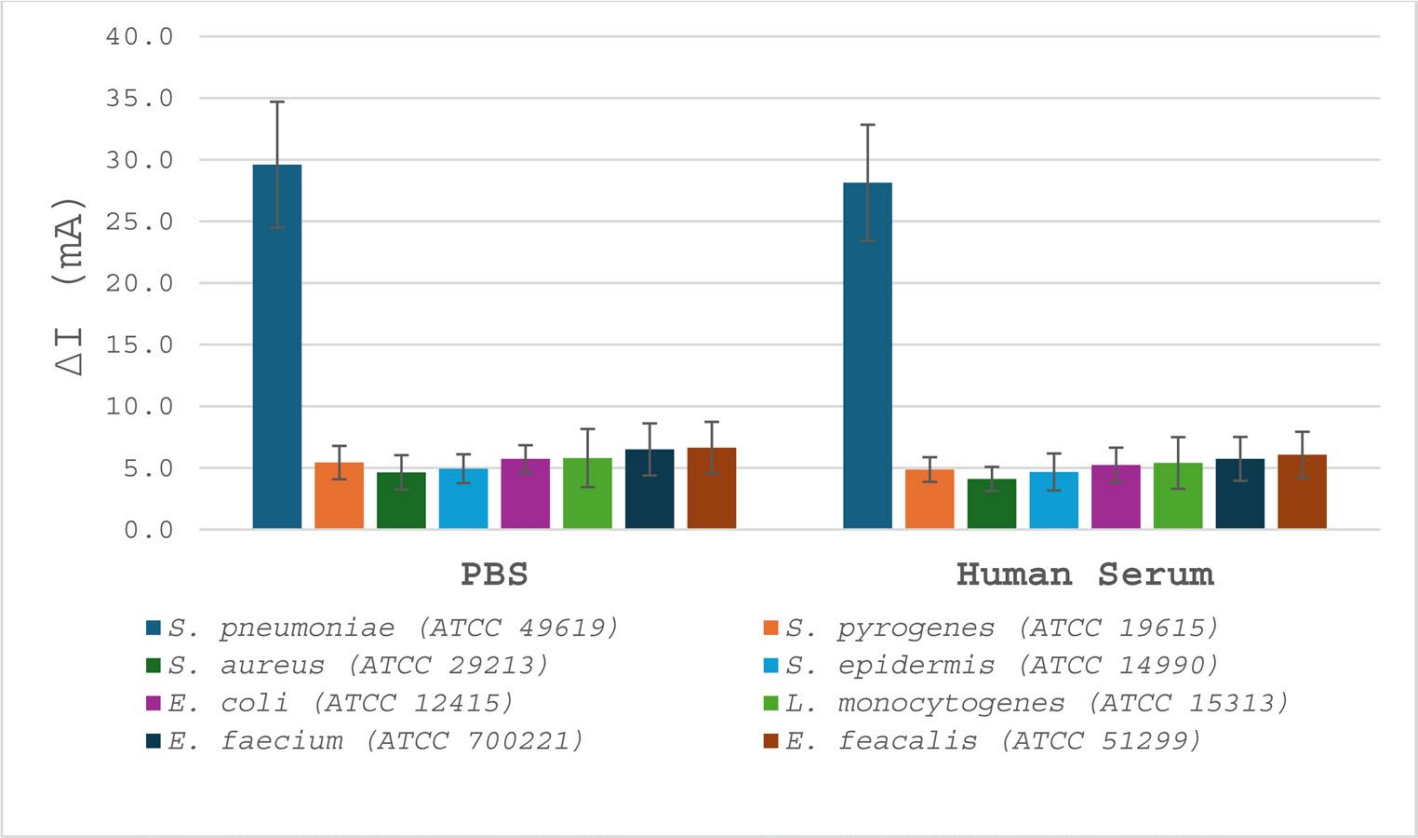
Selectivity of oligonucleotide-decorated hybrid nanoparticles toward *S. pneumoniae* in PBS and human serum. The SWV signal response (ΔI, µA) obtained at a bacterial concentration of 10³ CFU mL⁻¹ shows a pronounced increase for *S. pneumoniae* (ATCC 49619) in both media, whereas the non-target strains *S. pyogenes*, *S. aureus*, *S. epidermidis*, *E. coli*, *L. monocytogenes*, *E. faecium*, and *E. faecalis* exhibit only minimal responses. Data are presented as mean ± standard deviation from three independent experiments (*n* = 3). For the PBS dataset, one-way ANOVA followed by Tukey’s multiple-comparison test confirmed that *S. pneumoniae* differed significantly from all non-target strains (*p* < 0.0001)

The experimental procedures were adapted from previously established protocols [10, 21]. *S. pneumoniae* cells were cultured on tryptone soy agar supplemented with 5% defibrinated sheep blood (Thermo Scientific, Inc., Waltham, MA, USA) and incubated overnight at 37 °C. Bacterial suspensions equivalent to 0.5 McFarland standard were prepared in Tryptic Soy Broth (TSB). From each suspension, 1 mL was centrifuged, and the resulting pellets were resuspended in 100 µL of phosphate-buffered saline (PBS) or human serum (Sigma-Aldrich, Germany) to be used as cell suspension samples (Figs. 2, 3, 4 and 5). Human-serum measurements were included as a preliminary matrix-tolerance assessment for the selectivity experiments shown in Fig. 6. The screening protocol was conducted as previously described (Fig. 2A).

### Electrochemical analysis

Screen-printed gold electrodes (SPCEs) were utilized in conjunction with the microfluidic magnetic adaptor of the Dropsens i-Stat 400 potentiostat (DRP-CFLWCL-MAGN, Metrohm) (Fig. 2B). A total of 200 µL of each sample, prepared in 0.9% NaCl solution, was introduced into the microfluidic inlet at a constant flow rate of 10 µL/min. Electrochemical readings were recorded immediately following sample injection. Cyclic voltammetry (CV) and square-wave voltammetry (SWV) were performed sequentially for each sample. CV analyses were conducted over a potential range of − 0.4 V to 0.8 V with a scan rate of 50 mV s⁻¹. SWV measurements were performed within the potential range of 0.4 V to 0.8 V. Data acquisition and analysis were performed using DropSens 8400 software (version 3.6, 20B0514). For electrochemical control experiments, SPION/POSS (probe-free nanoparticle control), oligo-SPION/POSS without bacterial exposure (intact probe-coated nanoparticle control), and free Fc-labelled probe alone were also measured under the same instrumental conditions. The detection limit was estimated from the calibration plot in Fig. 5D using simple linear regression of current versus log(CFU mL⁻¹).

### Statistical analysis

Data are presented as mean ± standard deviation from three independent experiments (*n* = 3). Selectivity data shown in Fig. 6 were analyzed by one-way ANOVA followed by Tukey’s multiple-comparison test, and differences were considered statistically significant at *p* < 0.05.

## Results and discussion

### Greener synthesis and construction of nuclease-responsive magnetic nanohybrids

We aimed to use a stable and biocompatible surface for best signal production with nuclease responsive probes. Compared to conventional synthesis routes that require higher temperatures, or harsher organic solvents, the method described here can be considered greener due to its short reaction time, mild conditions, and reduced solvent consumption. PDA@SPION/POSS particles have advantages originating from their synthesis procedure. A stepwise fabrication strategy was successfully employed to construct oligonucleotide-functionalized PDA@SPION/POSS hybrid nanoparticles. The POSS-based organic–inorganic hybrid nanoparticles were synthesized via a UV-initiated emulsion polymerization using a green synthesis approach, yielding uniformly spherical particles within 5 min (Fig. 3A) [22]. Subsequently, in situ deposition of superparamagnetic iron oxide nanoparticles (SPIONs) [20, 22], followed by polydopamine (PDA) coating [20], was carried out to produce SPION/POSS and PDA@SPION/POSS hybrid structures, respectively. Scanning electron microscopy (SEM) images (Fig. 3A–D) reveal the morphological character of POSS nanoparticles in each step modification. The bare POSS nanoparticles exhibit a uniform spherical morphology with smooth surfaces, consistent with rapid UV-initiated emulsion polymerization (Fig. 3A). The absence of irregular aggregates or collapsed structures indicates effective stabilization during polymerization and confirms the suitability of this approach for producing submicron hybrid nanoparticles. Following in situ SPION deposition, the SPION/POSS nanoparticles (Fig. 3B) display a distinctly roughened surface texture.

This morphological change is attributed to the homogeneous nucleation and anchoring of Fe₃O₄ nanoparticles on the POSS matrix, rather than free SPION formation in solution. The preservation of overall particle shape at this stage suggests that the SPION decoration occurs on the surface of the nanoparticles without compromising the core. Subsequent polydopamine (PDA) coating further alters the surface topography, producing a granular and irregular morphology (Fig. 3C). This observation is characteristic of PDA layers formed via oxidative self-polymerization and provides strong qualitative evidence for successful PDA deposition. Importantly, oligonucleotide conjugation (Fig. 3D) does not induce noticeable changes in morphology, indicating that probe immobilization occurs as a thin molecular layer rather than a bulky coating that could hinder surface accessibility or electrochemical performance.

The hydrodynamic size of the nanoparticles was analyzed by dynamic light scattering (DLS), and the results are summarized in Table 1. The pristine POSS nanoparticles showed an average size of 111.5 nm with a PDI of 0.380. After SPION deposition, the particle size slightly increased to 113.2 nm, indicating successful surface decoration with iron oxide nanoparticles. Subsequent PDA coating and oligonucleotide conjugation resulted in gradual increases in hydrodynamic diameter to 117.3 nm and 120.8 nm, respectively. These size increments are consistent with the stepwise surface functionalization observed in the SEM images presented in Fig. 3, confirming the successful construction of the hybrid nanostructures.

**Table 1:** The dynamic light scattering (DLS) results

Nanoparticle name	Size(nm)	St. Dev. (±)	PDI
POSS	111.5	63.83	0.380
SPION/POSS	113.2	70.09	0.452
PDA@SPION/POSS	117.3	50.96	0.400
Oligo-SPION/POSS	120.8	55.18	0.323

EDX analysis confirms the stepwise surface functionalization of the nanoparticles (Fig. 3; Table 2). POSS nanoparticles show dominant silicon and oxygen signals, characteristic of the silsesquioxane cage, with a moderate carbon contribution from the methacrylate framework. Following SPION incorporation, a strong iron signal (17.60 wt%) appears, verifying successful magnetite deposition on the POSS surface. The accompanying decrease in silicon content indicates surface coverage by iron oxide nanoparticles. Subsequent PDA coating and oligonucleotide immobilization lead to a substantial increase in carbon content, reaching 70.62 wt% for oligo-SPION/POSS nanoparticles. In parallel, silicon and iron signals decrease markedly, consistent with the formation of a dense organic overlayer. This is further supported by atomic percentage data, where silicon drops from 19.92% (POSS) to 1.17% (probe-SPION/POSS), demonstrating effective surface coverage and the formation of an organic-rich interface favourable for biosensing applications.

**Table 2:** Energy-dispersive X-ray spectroscopy (EDX) results

NanoparticlesName	Element (%)							
	C		O		Si		Fe	
	Wt.	At.	Wt.	At.	Wt.	At.	Wt.	At.
POSS	44.65	58.81	23.54	23.27	31.81	19.92	-	-
SPION/POSS	41.76	59.98	20.75	22.38	19.88	12.21	17.60	5.44
PDA@SPION/POSS	48.44	61.72	30.05	28.74	13.43	7.32	8.08	2.22
Probe-SPION/POSS	70.62	77.52	25.45	20.37	2.50	1.17	1.44	0.32

FTIR spectroscopy further corroborates the stepwise functionalization process (Fig. 4A). All nanoparticle formulations retain the characteristic Si–O–Si stretching vibration around 1100 cm⁻¹ and Si–C vibrations near 760 cm⁻¹, confirming that the POSS framework remains chemically intact throughout synthesis and modification [23]. The appearance of Fe–O stretching bands around 580 cm⁻¹ in SPION/POSS nanoparticles verifies magnetite formation^21^. After PDA coating, new absorption bands emerge in the 1500–1650 cm⁻¹ region, attributed to aromatic C = C and N–H bending vibrations, along with a broad band between 3200 and 3500 cm⁻¹ corresponding to O–H and N–H stretching^24^. These features are hallmarks of PDA chemistry and confirm successful mussel-inspired surface functionalization, which is essential for robust oligonucleotide immobilization.

X-ray diffraction (XRD) analysis (Fig. 4B) demonstrates the coexistence of amorphous polymeric and crystalline inorganic phases within the hybrid nanoparticles. Bare POSS nanoparticles exhibit a broad amorphous halo centered at approximately 22°, typical of polymeric siloxane networks. In contrast, SPION/POSS nanoparticles display distinct diffraction peaks at 2θ values corresponding to the (220), (311), (400), (422), (511), and (440) planes of magnetite (Fe₃O₄), in agreement with JCPDS No. 19-062921. The retention of these peaks after surface modification confirms that the SPIONs maintain their crystalline structure and are not chemically altered during PDA coating. The simultaneous presence of amorphous and crystalline features confirms the successful fabrication of a true organic–inorganic hybrid system.

Vibrating sample magnetometry (VSM) measurements (Fig. 4C) reveal superparamagnetic behaviour for both SPION/POSS and PDA@SPION/POSS nanoparticles, as evidenced by negligible coercivity and remanence. This property is essential for biosensing applications, as it allows rapid magnetic collection and electrode localization. The saturation magnetization (Ms) decreased from approximately 10 emu/g (SPION/POSS) to 8 emu/g (PDA@SPION/POSS) after PDA coating, Importantly, this magnetic responsiveness was sufficient for rapid magnetic separation during biosensing, ensuring facile particle manipulation without residual magnetization effects.

Although a dedicated long-term storage stability study was beyond the scope of the present work, the combined DLS, FTIR, XRD, and VSM results indicate that PDA@SPION/POSS nanohybrids preserve their structural integrity and magnetic functionality throughout the surface-modification steps and under the assay conditions used here. Thus, the current data supports short-term functional stability of the nanohybrids during preparation and electrochemical measurement, while longer-term storage stability should be evaluated in future studies.

### Magneto-electrochemical performance and analytical sensitivity

The sensing mechanism is based on pathogen-associated nuclease activity. The Fc-labeled, nuclease-responsive oligonucleotide probe immobilized on PDA@SPION/POSS is cleaved upon exposure to *S. pneumoniae*, generating a measurable electrochemical response. Importantly, the MAEB configuration (microfluidic magnetic adaptor and SPCE) localizes the probe-bearing particles at the electrode surface, increasing effective local probe concentration and enabling rapid signal acquisition with reduced incubation demands.

Figure 5A (Cyclic Voltammetry-CV measurements) and Fig. 5B (SWV) provide complementary evidence that the Fc-associated electrochemical signal changes in the presence of *S. pneumoniae*. In practice, SWV is particularly well suited for surface-confined redox reporting because it enhances sensitivity to interfacial electron-transfer changes, making it appropriate for detecting cleavage-dependent reconfiguration of Fc-containing probes on magnetically localized nanoparticles. The agreement between CV/SWV responses supports that the observed signal modulation is electrochemically consistent and not an artifact of a single measurement mode.

In the intact oligo-SPION/POSS construct, the surface-bound Fc-labelled oligonucleotide layer partially passivates the magnetically localized nanoparticle/electrode interface and lowers the SWV current relative to probe-free SPION/POSS. After exposure to *S. pneumoniae*, nuclease-mediated cleavage partially removes this interfacial oligonucleotide layer, thereby relieving surface blocking and producing a higher current than the bacteria-free oligo-SPION/POSS control, as observed in Fig. 5B. Thus, the analytical readout is a cleavage-induced current recovery relative to the intact oligo-SPION/POSS control rather than a signal generated by freely diffusing cleaved Fc fragments. Control experiments with free Fc-labelled probe alone produced a response comparable to the bare gold electrode (285 ± 63 µA vs. 278 ± 72 µA), further indicating that the measurable response originates from probe-functionalized magnetic nanohybrids.

Figure 5A and B also clarify the role of the electrochemical controls. SPION/POSS serves as the probe-free nanoparticle background, whereas oligo-SPION/POSS serves as the intact probe-coated control in the absence of bacteria. The partial signal recovery observed after exposure to *S. pneumoniae* is therefore consistent with nuclease cleavage of the immobilized oligonucleotide layer. In this context, the MAEB configuration concentrates the nanoparticle layer at the electrode surface, shortens transport distances, and enables rapid electrochemical interrogation within minutes. Compared with the earlier planar-electrode Fc-P3 format [10], the present MAEB workflow yields a measurable response within only a few minutes of sample preparation. Because the two formats are not identical, this observation should be interpreted as incubation-time optimization within the current magnetic format rather than as a strict head-to-head time comparison.

Figure 5C examines how the initial amount of hybrid nanoparticles affects signal response using a representative bacterial concentration (10⁵ CFU mL⁻¹). This optimization is essential because particle dose governs two opposing factors: (i) Probe reservoir and signal amplitude and (ii) accessibility and transport constraints. Increasing particle amount increases the total Fc-probe amount delivered to the electrode region, typically improving baseline signal strength and dynamic response potential. Excessive particle loading can overcrowd the electrode zone, increase diffusion barriers (for nucleases and ions) and potentially limit effective electron transfer to probes. The presence of a dose–response dependence in Fig. 5C indicates that assay performance can be systematically tuned by balancing probe density against interfacial accessibility.

The platform shows wide-range detection from 10^2^ to 10⁸ CFU mL⁻¹, with a reported detection limit of 10^2^ ± 21 CFU mL⁻¹ (Fig. 5D). This broad dynamic range is operationally valuable because pneumococcal burdens in clinical and surveillance contexts can vary substantially depending on sample type and disease stage. The sensitivity is particularly noteworthy because the approach does not rely on nucleic acid replication or PCR-based amplification. Instead, signal enhancement arises from the intrinsic catalytic activity of bacterial nucleases, where enzymatic turnover of multiple oligonucleotides generates amplified electrochemical response when coupled to magnetic enrichment and surface-confined detection.

This high selectivity arises from the nuclease-specific oligonucleotide design, which is preferentially cleaved by pneumococcal nucleases while remaining largely intact in the presence of other bacterial species. In contrast to affinity-based sensors such as antibody platforms, where signal generation is typically limited to a one-to-one recognition event, nuclease-driven detection benefits from enzymatic turnover, enabling a single active enzyme to cleave multiple probe molecules. This catalytic mechanism provides intrinsic signal amplification while maintaining biological specificity, offering a distinct conceptual advantage over purely binding-based detection strategies [7, 9].

The results are in strong agreement with earlier studies demonstrating that nuclease-activated probes enable highly specific detection of pathogenic bacteria both in vitro and in complex biological matrices [10, 15, 16]. Notably, the clear signal separation between *S. pneumoniae* and all control strains at a clinically relevant concentration (10³ CFU mL⁻¹) highlights the robustness of the sensing strategy and its potential applicability in real diagnostic settings. The combination of high sensitivity (Fig. 5) and excellent selectivity (Fig. 6) positions this platform as a competitive alternative to existing electrochemical and microfluidic biosensors for pneumococcal detection, particularly in contexts where rapid, label-free, and low-cost diagnostics are required.

The selectivity of the biosensing platform was evaluated in both PBS and human serum against a panel of clinically relevant bacterial strains, as shown in Fig. 6. In PBS, *S. pneumoniae* produced a pronounced SWV signal change (ΔI = 29.6 ± 5.1 µA), whereas the non-target strains yielded only minimal responses (4.6–6.6 µA). A similar discrimination pattern was retained in human serum, where *S. pneumoniae* gave 28.1 ± 4.7 µA and the control strains remained in the 4.1–6.1 µA range, indicating limited matrix interference under the tested conditions. For the PBS dataset, one-way ANOVA confirmed a significant overall difference among the tested strains (F(7,16) = 36.76, *p* = 1.07 × 10⁻⁸). Tukey’s multiple-comparison test showed that the SWV response for *S. pneumoniae* was significantly higher than that of each non-target strain (all adjusted *p* < 0.0001), whereas no significant differences were observed among the control strains (*p* > 0.05).

Notably, the clear signal separation between *S. pneumoniae* and all control strains at a clinically relevant concentration (10³ CFU mL⁻¹) highlights the robustness of the sensing strategy and its potential applicability in real diagnostic settings. The combination of high sensitivity (Fig. 5) and excellent selectivity (Fig. 6) positions this platform as a competitive alternative to existing electrochemical and microfluidic biosensors for pneumococcal detection, particularly in contexts where rapid, label-free, and low-cost diagnostics are required.

Figure 5E therefore presents the incubation-time dependence of the current MAEB workflow. This experiment should not be interpreted as a direct replication of the previously published planar-electrode assay under identical conditions, because the earlier study used surface-immobilized Fc-P3 on gold electrodes whereas the present platform uses PDA@SPION/POSS-supported probes under magnetic confinement [10]. Nevertheless, the data show that the MAEB configuration generates a measurable response within the short incubation windows tested here, which is consistent with the expected benefits of mobile probe carriers, magnetic enrichment, and immediate electrochemical readout.

The analytical performance of the proposed MAEB system was compared with representative *S. pneumoniae* biosensing strategies, as provided in Table 3. Relative to the disposable antibody-based magnetoimmunosensor reported by Campuzano et al. [6], the present platform provides substantially improved whole-cell sensitivity. Compared with the aptamer-assisted microfluidic electrochemical platform of Babaie et al. [21], the present system also shows a lower bacterial detection limit and a broader working range. Importantly, in comparison with the previously reported planar Fc-P3 electrochemical assay using the same nuclease-responsive probe chemistry [10], the present MAEB format preserves the high analytical sensitivity while adding magnetic enrichment and nanoparticle-supported probe presentation, which are operationally advantageous for disposable electrode integration. Because assay format, target type, and sample matrix differ among reports, this comparison should be interpreted as cross-study and indicative rather than as a strict head-to-head ranking. Collectively, these features position the MAEB platform as a sensitive and operationally simple alternative for rapid pneumococcal screening.

**Table 3:** Comparison of the present MAEB biosensor with representative *S. pneumoniae* detection methods

Method	Target/ recognition principle	Readout format	Analytical performance	Main limitation	Advantage of present study
[6]	Whole-cell antibody recognition	Disposable amperometric magnetoimmunosensor	LOD: 1.5 × 10⁴ CFU mL⁻¹ for strain Dawn LOD: 10⁵ CFU mL⁻¹ for strain R6	Antibody-based format and markedly higher whole-cell LOD	Present MAEB gives lower bacterial LOD and uses catalytic nuclease readout rather than antibody sandwich recognition
[10]	Membrane-associated nuclease activity using Fc-P3 on planar gold electrode	Electrochemical (CV/SWV)	LOD: 10² CFU mL⁻¹; short probe-bacteria contact times of 5–60 min were evaluated	Planar gold-electrode immobilization; no magnetic enrichment	Present MAEB retains comparable sensitivity while adding magnetic concentration and particle-supported probe handling
[21]	Whole-cell aptamer capture	Microfluidic isolation + electrochemical electrode array	Isolation ~ 1 min; LOD: 962 CFU mL⁻¹; linear range: 10⁴–10⁷ CFU mL⁻¹	Requires aptamer capture architecture and separate isolation/detection design	Present MAEB shows lower bacterial LOD and broader dynamic range
Present study	Whole-cell pathogen-associated nuclease activity on PDA@SPION/POSS nanohybrids	Magneto-electrochemical SWV on disposable SPCE	LOD: 10² CFU mL⁻¹; dynamic range: 10²–10⁸ CFU mL⁻¹	—	Combines high sensitivity, magnetic enrichment, amplification-free whole-cell detection, and green nanohybrid synthesis

## Conclusion

This work presents a sustainable magneto-electrochemical biosensing platform for selective detection of *S. pneumoniae* based on pathogen-associated nuclease activity. Uniform organic–inorganic POSS nanoparticles were synthesized via ultrafast UV-initiated emulsion polymerization (5 min) and subsequently converted into functional biosensing nanohybrids through in situ SPION decoration and biomimetic PDA coating, enabling robust thiol-oligonucleotide immobilization while preserving structural integrity. Physicochemical characterization (SEM/DLS/EDX/FTIR/XRD/VSM) confirms controlled particle growth, effective stepwise surface coverage, and superparamagnetic behavior suitable for rapid magnetic manipulation and electrode localization.

Electrochemical measurements demonstrate nuclease-triggered signal generation, where intrinsic enzymatic turnover provides catalytic signal amplification without nucleic acid replication, enabling sensitive detection across 10²–10⁸ CFU mL⁻¹ with a detection limit of 10² CFU mL⁻¹, alongside excellent selectivity against multiple non-target bacterial strains. Preliminary experiments in human serum further retained clear discrimination of *S. pneumoniae* over the control strains, indicating initial compatibility with a clinically relevant matrix. Compared with the previously reported planar-electrode Fc-probe format [10], the present magnetic probe-particle MAEB strategy provides robust signal generation after short incubation periods within the current workflow (Fig. 5E). Because the two formats are not identical, this should be interpreted as a platform-level improvement rather than a strict head-to-head time comparison.

Overall, the PDA@SPION/POSS nanohybrids provide a green, scalable materials route and a practical diagnostic workflow with strong potential for rapid pneumococcal screening. Future work should focus on expanded validation in clinically relevant matrices, robustness against sample variability, and integration into streamlined sample-to-answer formats for point-of-care deployment. Future work should also address long-term colloidal and storage stability of PDA@SPION/POSS nanohybrids under practical handling conditions.

## Data Availability

Data is available upon reasonable request.
